# Tableau-based protein substructure search using quadratic programming

**DOI:** 10.1186/1471-2105-10-153

**Published:** 2009-05-19

**Authors:** Alex Stivala, Anthony Wirth, Peter J Stuckey

**Affiliations:** 1 Department of Computer Science and Software Engineering, The University of Melbourne, Melbourne, Victoria 3010, Australia; 2 NICTA Victoria Laboratories at The University of Melbourne, Melbourne, Victoria, Australia

## Abstract

**Background:**

Searching for proteins that contain similar substructures is an important task in structural biology. The exact solution of most formulations of this problem, including a recently published method based on tableaux, is too slow for practical use in scanning a large database.

**Results:**

We developed an improved method for detecting substructural similarities in proteins using tableaux. Tableaux are compared efficiently by solving the quadratic program (QP) corresponding to the quadratic integer program (QIP) formulation of the extraction of maximally-similar tableaux. We compare the accuracy of the method in classifying protein folds with some existing techniques.

**Conclusion:**

We find that including constraints based on the separation of secondary structure elements increases the accuracy of protein structure search using maximally-similar subtableau extraction, to a level where it has comparable or superior accuracy to existing techniques. We demonstrate that our implementation is able to search a structural database in a matter of hours on a standard PC.

## Background

Finding structures in a database which contain a substructure that is similar to a query structure or structural motif is an important technique in analyzing protein structure, function, and evolution. There are many existing methods for finding structurally similar proteins which take diverse approaches, such as: structural alignment at the level of residues or backbone atoms [1,2] or (as an initial step) secondary structure elements [3-7], purely topological matching [8,9], and probabilistic approaches [10-12]. Detailed structural alignment, however, although capable of great accuracy, is often slow [2], and therefore impractical for searching entire databases of the size of SCOP [13,14] or the PDB [15].

The TOPS-based method [8,9] provides structural motif searches, but by operating purely on topology it "ignores other important spatial properties" [[16], p. 1331]. Non-alignment approaches, such as PRIDE [10], can be extremely fast, but not as accurate as alignment-based approaches [17], and provide only a matching score, not an alignment or a coarse-grained or seed alignment for further refinement.

Two recent approaches, ProSMoS [16] and TableauSearch [18], use spatial interactions between secondary structure elements (SSEs) to find common structures. ProSMoS constructs a "meta-matrix" of SSEs and the interactions between them, and finds all possible submatrices in a database meta-matrix that match the query meta-matrix. TableauSearch constructs tableaux [19,20], which represent relative orientations of SSEs, and finds substructural matches by extracting maximally-similar subtableaux between the query tableau and a database tableau. In the exact (rigorous) technique, this problem is expressed as a quadratic integer program (QIP) or integer linear program (ILP) [18] and solved exactly using ILOG CPLEX [21]. Both ProSMoS and the exact tableau search formulation allow substructures to be found within structures. They also allow non-linear matchings, that is, sets of correspondences between SSEs in which the sequential order of corresponding SSEs is not preserved. Such non-linear matchings have recently been shown to be significantly more widespread than had previously been thought [22], and are therefore of considerable interest.

The two most similar methods to tableau matching are perhaps LOCK [5], and its newer version LOCK 2 [6], and ProSMoS. LOCK and LOCK 2 also match SSE vectors between structures, but use a more complex set of seven scoring functions, both orientation dependent and orientation independent, and use iterative dynamic programming requiring parameters for each of the scoring functions [5]. In contrast, the tableau matching formulation is simpler and more elegant, although to obtain higher accuracy we extend it with a distance difference constraint that requires a parameter.

ProSMoS, although it is similar to tableau matching in its use of SSE orientations, takes quite a different approach from most existing structural search methods in that, rather than taking a structure (or substructure) definition as a query, the query meta-matrix is constructed manually (or at least modified manually from one generated by the supplied scripts) by the user. This is clearly useful for finding user-specified motifs in a database of structures, but creates challenges in assessing the performance of the method since the results are so dependent on the user-specified query. ProSMoS, in contrast to our method, returns a list of hits to the query structure, rather than a matching score for each structure in the database. This is often simpler for the user, but it does have the disadvantage that finding more (or fewer) hits requires editing the query meta-matrix, which can be quite difficult to calibrate. Returning a score for each database structure means that adjusting the sensitivity or specificity required is simply a matter of varying the cutoff score for a match to be considered significant.

An advantage of the maximally-similar subtableaux formulation is that it allows the discovery of similar substructures within two structures, without requiring that the two structures are themselves similar as a whole, or that one of the structures must match as a whole some substructure within the other structure. We may choose to use one structure as a "query" motif, usually a small well-defined structural folding pattern, and find structures that contain this entire motif as a substructure, but it is also possible to find common substructures in two unrelated folds.

However, the rigorous tableau searching method is too slow for a full database search, and so Konagurthu *et al*. [18] introduce TableauSearch. This method approximates the exact solution using an alignment-like approach [23], with two phases of dynamic programming. TableauSearch is extremely fast, but loses the rigorous theoretical justification and is not as accurate as the exact method. It is also inherently sequential, thereby losing the ability to find non-linear structural matchings, and, at least partly, loses the ability to find substructural (local) rather than full structure (global) matches. This may be possible, however, by removing end gap penalties [18].

Here we present a method, based on the exact tableaux matching formulation [18] and recent work in alignment of molecular networks [24], that allows searches for occurrences of a query structure as substructures of structures in a database such as SCOP in practical time, allows non-linear matchings, and is able to provide a set of correspondences between SSEs.

## Results and discussion

We evaluate the accuracy of our QP tableau matching algorithm as a method for determining the fold of a structure, using SCOP as the truth. The tradeoff between sensitivity and specificity for such a classification task can be shown as a Receiver Operating Characteristic (ROC) curve [25].

**Figure 1:**
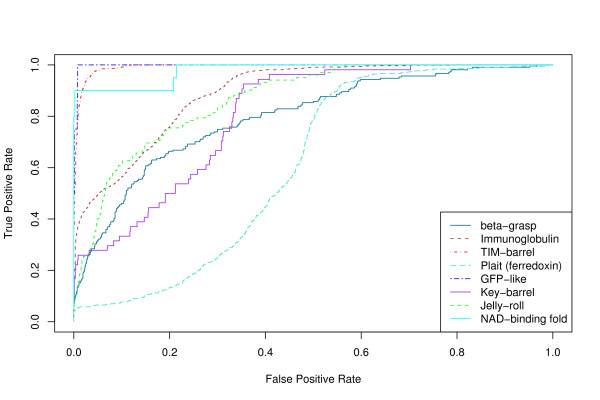
**ROC curves without using distance information**. ROC curves for the structural folding patterns in Table 1 as the query structure, using the discrete tableau encoding and no distance information. A true positive is a "hit" on a structure that is in the same SCOP fold as the query.

**Figure 2:**
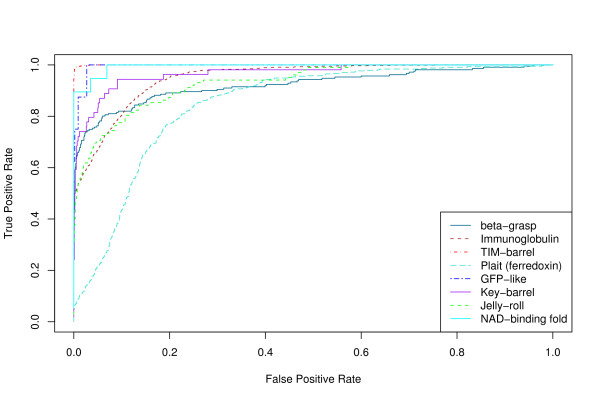
**ROC curves using distance information**. ROC curves for the structural folding patterns in Table 1 as the query structure, using the discrete tableau encoding and distance information. A true positive is a "hit" on a structure that is in the same SCOP fold as the query.

ROC curves for several different structural queries are shown in Figures 1 and 2. Figure 1 shows results obtained using only tableau information, while Figure 2 shows results obtained using tableau and distance information (see Methods). The area under the ROC curve (AUC – see Methods) for each of these curves is shown in Table 1. Incorporating distance information results in consistently higher AUC values without significantly affecting the elapsed time.

**Table 1:** AUC and time for some widespread folds.

			distance information			
			without		with	
Fold	SCOP sid	# SSEs	AUC	time	AUC	time
*β*-grasp	d1ubia_	8	0.80	0 h 47 m	0.92	0 h 32 m
Key-barrel	d1tttb1	9	0.80	0 h 50 m	0.97	0 h 50 m
Immunoglobulin	d1ae6h1	13	0.89	1 h 47 m	0.95	1 h 53 m
Plait (ferredoxin)	d1bhne_	15	0.61	1 h 53 m	0.85	2 h 18 m
GFP-like	d1h6rb_	17	1.00	3 h 06 m	0.99	3 h 06 m
Jelly-roll	d2phlb1	19	0.87	4 h 24 m	0.93	5 h 13 m
Tim-barrel	d1tima_	21	0.99	4 h 31 m	1.00	4 h 30 m
NAD-binding fold	d1f6dc_	30	0.98	14 h 45 m	0.99	16 h 33 m

Area under ROC curve (AUC) and time taken for searches for several structural folding patterns against the ASTRAL SCOP 95% sequence identity non-redundant database consisting of 15273 domains using discrete tableaux, both with and without incorporating SSE distance information. Times (in hours and minutes) reported are elapsed times using the sparse matrix (UMFPACK) implementation on an Intel Xeon 3.2 GHz machine with 8 GB memory running Linux. The table is sorted by query size (number of SSEs in query).

We find that the best-performing variation of our method, using the discrete tableau encoding rather than numeric Ω matrices, and incorporating distance information, has an AUC of 0.95 averaged over the eight queries in Table 1.

We can see in Table 1 that the ferredoxin fold query (d1bhne_) performs significantly worse than the others. We examined the results from this query and found that a large number of false negatives occur (many members of this fold are not given a high score by our method). Examining some of these false negatives in detail, we find that it is often due to DSSP [26], which we use to define SSEs, not defining some of the SSEs required to match the query structure (in an extreme case, d2atcb1, DSSP defines only a single helix and nothing else). Although we have the capability of using STRIDE [27] rather than DSSP, the results are often similar (as in the d2atcb1 example). This is a shortcoming of any method that depends on SSE definitions, although ProSMoS solves it to some degree by using PALSSE [28], a secondary structure assignment method that assigns many more residues to SSEs precisely in order to avoid this problem [16]. False negatives can also occur independently of the SSE definition algorithm, if a structure does not have some SSEs not considered essential to the fold according to SCOP (but which are included in our query structure) and/or sufficiently different in their orientation that tableau matching does not assign them a high score. An example of this is d1q8ba_, which does not have all the helices in the query structure, and some which it does include have rather different orientations from those in the query, but it is nevertheless classified as a member of the ferredoxin-like fold.

Table 1 shows that a search in a database of 15273 structures takes approximately one hour for a small (10 or fewer SSEs) query structure on a single CPU of a standard PC, and under four hours for query structures with fewer than 18 SSEs, but can take more than 16 hours for a query structure with 30 SSEs. Since 75% of domains in the database have fewer than 20 SSEs, and the most frequent number of SSEs is 10, most queries for a structure drawn from a set of structures with the same distribution of tableau size as the database will complete within 4 hours. We note that the peak at 10 differs from the results of [20], who find the peak is at 6, as we have used DSSP to define SSEs and have included *π*- and 3_10_-helices, while Kamat and Lesk [20] used the assignments of helices and strands from PDB files.

**Figure 3:**
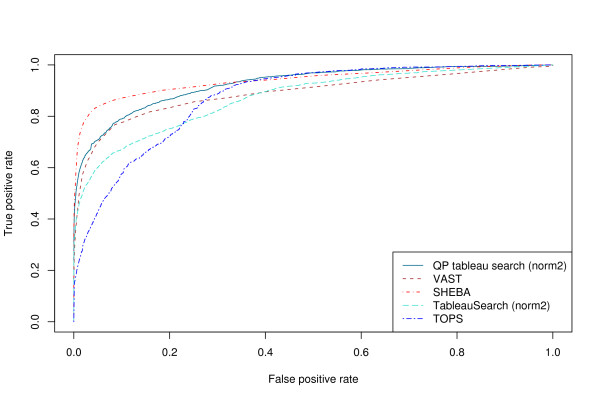
**ROC curves for different methods**. ROC curves for the 200 query set against the ASTRAL SCOP 95% sequence identity non-redundant database consisting of 15273 domains.

For a larger-scale test, we used a set of 200 queries chosen from the ASTRAL SCOP 1.73 95% sequence identity non-redundant data set [14,29]. The queries were chosen so that each class (*α*, *β*, *α*/*β*, *α *+ *β*) is represented in the query set in the same ratio as it is in the database. The overall AUC for different normalizations (see Methods) and different methods are shown in Table 2. It can be seen that normalization norm2 is the best normalization function for the tableau search methods, and that SHEBA [30] is the best performing method, followed by our method, then VAST [3,4], and lastly TableauSearch [18] and TOPS. Figure 3 shows the ROC curves for the different methods (using the best normalization function where appropriate). It can be seen that the curve for SHEBA lies above that for VAST at every point, consistent with the results for all-against-all comparisons in the ASTRAL SCOP 1.63 40% sequence identity non-redundant data set reported in [25]. The curve for our method lies between the two at low false positive rates, but crosses over the SHEBA curve at a false positive rate of approximately 0.4, indicating it has a slightly higher sensitivity at high false positive rates than SHEBA. SHEBA, however, is more sensitive at low false positive rates, a generally more useful attribute.

**Table 2:** AUC for the 200 query set.

Method	Normalization	AUC
SHEBA	None	0.941
QP tableau search	norm2	0.925
QP tableau search	norm1	0.904
QP tableau search	norm3	0.904
VAST	None	0.890
TableauSearch	norm2	0.871
TOPS	None	0.871
TableauSearch	norm1	0.869
QP tableau search	None	0.854
TableauSearch	None	0.846
TableauSearch	norm3	0.832

Area under ROC curve (AUC) for the 200 query set against the ASTRAL SCOP 95% sequence identity non-redundant database consisting of 15273 domains, for different methods and normalizations. Note that only the tableau search methods (our method (QP tableau search) and TableauSearch) require normalization, as VAST, SHEBA and TOPS report normalized scores themselves and so do not require an external normalization step to combine results for different queries. The table is ordered by decreasing AUC.

In terms of elapsed time (for a single processor core), TableauSearch is by far the fastest method. On our system, it has a total elapsed time for the 200 query set of only 1 hour 25 minutes, compared to 28 hours for VAST, 52 hours for SHEBA, and 741 hours for our method. Large scale comparisons with the exact solution of the QIP or ILP with CPLEX are not practical, as a single comparison takes at least several seconds and can take up to several days, and in some cases exhausts the virtual memory of our machine.

### Comparison with MAX-CMO heuristic

Maximum Contact Map Overlap (MAX-CMO) is a formulation of the problem of finding the similarity of two protein structures. MAX-CMO uses the contact map representation of proteins, in which a protein with *n *residues is represented as a square symmetric matrix *C*_*n *× *n *_where *C*_*ij *_= 1 when the distance between residues *i *and *j *is less than some threshold, and *C*_*ij *_= 0 otherwise. Typically this distance is defined as the C_*α *_distance, and the threshold is for example 7 Å. The MAX-CMO problem is then to find a (non-crossing) alignment of residues that maximizes the overlap between two contact maps. The value (or score) of the alignment is the number of contacts in one protein whose residues are aligned with residues that are also in contact in the other protein [31].

MAX-CMO is an NP-hard problem, and methods for solving it exactly, by such techniques as integer programming with Lagrangian relaxation [31,32] or branch-and-bound [33] can be impractically slow.

Therefore, heuristic approaches are useful, and recently a variable neighborhood search (VNS) algorithm for approximating MAX-CMO has been published, with an analysis of its effectiveness in ranking protein similarity [34].

Here we compare the performance of the QP formulation of maximally-similar subtableaux extraction with the VNS heuristic for MAX-CMO of [34].

**Figure 4:**
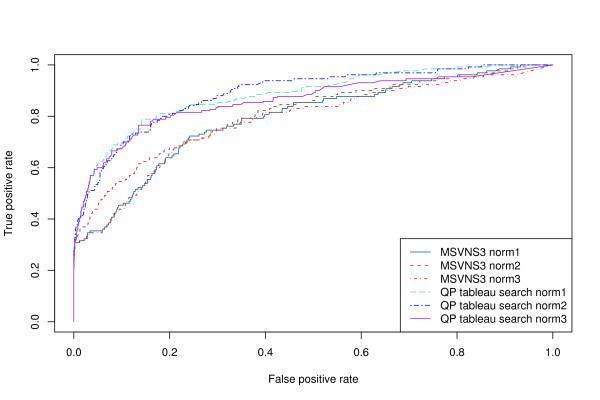
**ROC curves on the Fischer data set at fold level**. ROC curves for MSVNS3 and QP tableau search with different normalization functions on the Fischer data set at fold level.

**Figure 5:**
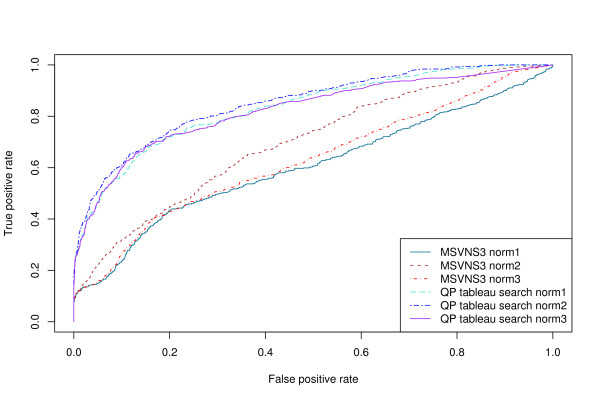
**ROC curves on the Fischer data set at class level**. ROC curves for MSVNS3 and QP tableau search with different normalization functions on the Fischer data set at class level.

Figure 4 shows the ROC curves for the Fischer data set [35] at the fold level, and Table 3 shows the corresponding AUC values. Figure 5 and Table 4 show the corresponding results at the class level. It is apparent that for the Fischer data set, the QP tableau search method achieves significantly higher accuracy at both levels than the MSVNS3 method, regardless of normalization type.

**Table 3:** Area under the ROC curve (AUC) for the Fischer data set at fold level.

				95% confidence interval	
Method	Normalization	AUC	standard error	lower	upper
MSVNS3	None	0.788	0.017	0.754	0.821
MSVNS3	norm1	0.791	0.017	0.758	0.824
MSVNS3	norm2	0.809	0.016	0.777	0.842
MSVNS3	norm3	0.781	0.017	0.747	0.815
QP tableau search	None	0.837	0.016	0.807	0.868
QP tableau search	norm1	0.882	0.014	0.855	0.909
QP tableau search	norm2	**0.887**	0.014	0.861	0.914
QP tableau search	norm3	0.860	0.015	0.831	0.889

**Table 4:** Area under the ROC curve (AUC) for the Fischer data set at class level.

				95% confidence interval	
Method	Normalization	AUC	standard error	lower	upper
MSVNS3	None	0.666	0.009	0.647	0.684
MSVNS3	norm1	0.604	0.010	0.586	0.623
MSVNS3	norm2	0.696	0.009	0.678	0.714
MSVNS3	norm3	0.628	0.010	0.610	0.647
QP tableau search	None	0.789	0.008	0.773	0.805
QP tableau search	norm1	0.833	0.008	0.819	0.848
QP tableau search	norm2	**0.851**	0.007	0.837	0.865
QP tableau search	norm3	0.824	0.008	0.809	0.839

**Figure 6:**
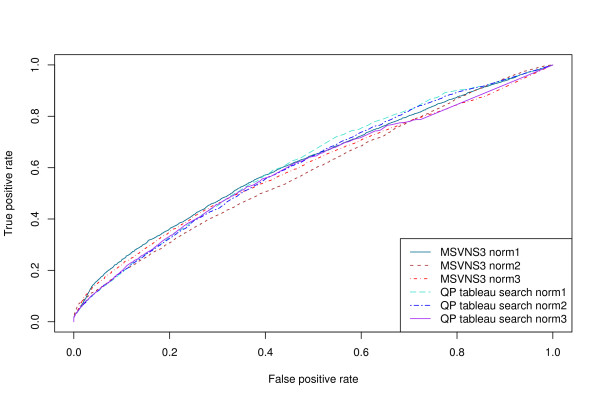
**ROC curves on the Nh3D data set at architecture level**. ROC curves for MSVNS3 and QP tableau search with different normalization functions on the Nh3D data set at architecture level.

**Figure 7:**
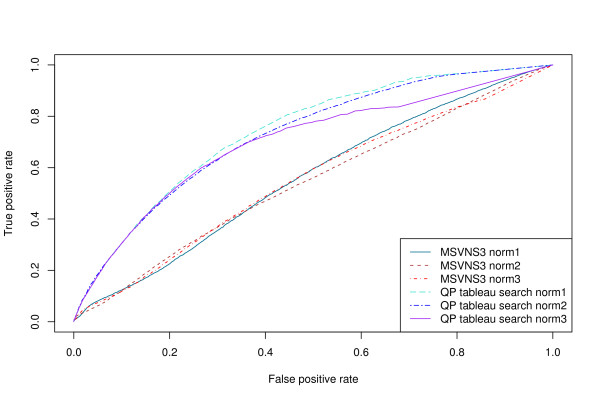
**ROC curves on the Nh3D data set at class level**. ROC curves for MSVNS3 and QP tableau search with different normalization functions on the Nh3D data set at class level.

Figure 6 shows the ROC curves for queries in the Nh3D data set [34,36] at the architecture level, and Table 5 shows the corresponding AUC values. Figure 7 and Table 6 show the corresponding results at the class level. At the architecture level, there is no significant difference in the performance of the two methods, but at the class level again QP tableau search has significantly higher accuracy when measured by AUC.

**Table 5:** Area under the ROC curve (AUC) for the Nh3D data set at architecture level.

				95% confidence interval	
Method	Normalization	AUC	standard error	lower	upper
MSVNS3	None	0.537	0.005	0.528	0.547
MSVNS3	norm1	**0.617**	0.005	0.607	0.627
MSVNS3	norm2	0.583	0.005	0.573	0.593
MSVNS3	norm3	0.598	0.005	0.588	0.608
QP tableau search	None	0.578	0.005	0.568	0.588
QP tableau search	norm1	**0.617**	0.005	0.607	0.626
QP tableau search	norm2	0.608	0.005	0.598	0.618
QP tableau search	norm3	0.599	0.005	0.589	0.608

**Table 6:** Area under the ROC curve (AUC) for the Nh3D data set at class level.

				95% confidence interval	
Method	Normalization	AUC	standard error	lower	upper
MSVNS3	None	0.590	0.003	0.585	0.595
MSVNS3	norm1	0.559	0.003	0.554	0.564
MSVNS3	norm2	0.543	0.003	0.538	0.548
MSVNS3	norm3	0.551	0.003	0.546	0.557
QP tableau search	None	0.708	0.002	0.703	0.712
QP tableau search	norm1	**0.740**	0.002	0.735	0.744
QP tableau search	norm2	0.726	0.002	0.722	0.731
QP tableau search	norm3	0.700	0.002	0.695	0.704

We should perhaps discount any superiority in the performance of the tableau search method at the class level, as this level of classification in the CATH hierarchy indicates only the percentage of *α*-helices and *β*-strands in the domain [37]. Since tableaux are based on SSEs (defined by DSSP) we could trivially obtain good classification performance at this level just from the DSSP classification, while MAX-CMO uses only residue contact information, and so must score protein similarity at this high level without having SSEs defined by an existing method.

Ignoring the class level comparisons therefore, we find that QP tableau search has significantly superior accuracy compared to MSVNS3 on the Fischer data set, and similar accuracy to MSVNS3 on the Nh3D data set.

For the Fischer data set, MSVNS3 took 8 hours while the sparse matrix (UMFPACK [38-41]) implementation of QP tableau search took 2 hours on a PC with an Intel Core 2 Duo processor and 2 GB of memory running 32-bit Linux. For the Nh3D data set, MSVNS3 took 62 hours while QP tableau search took 8 hours.

### Examples

**Figure 8:**
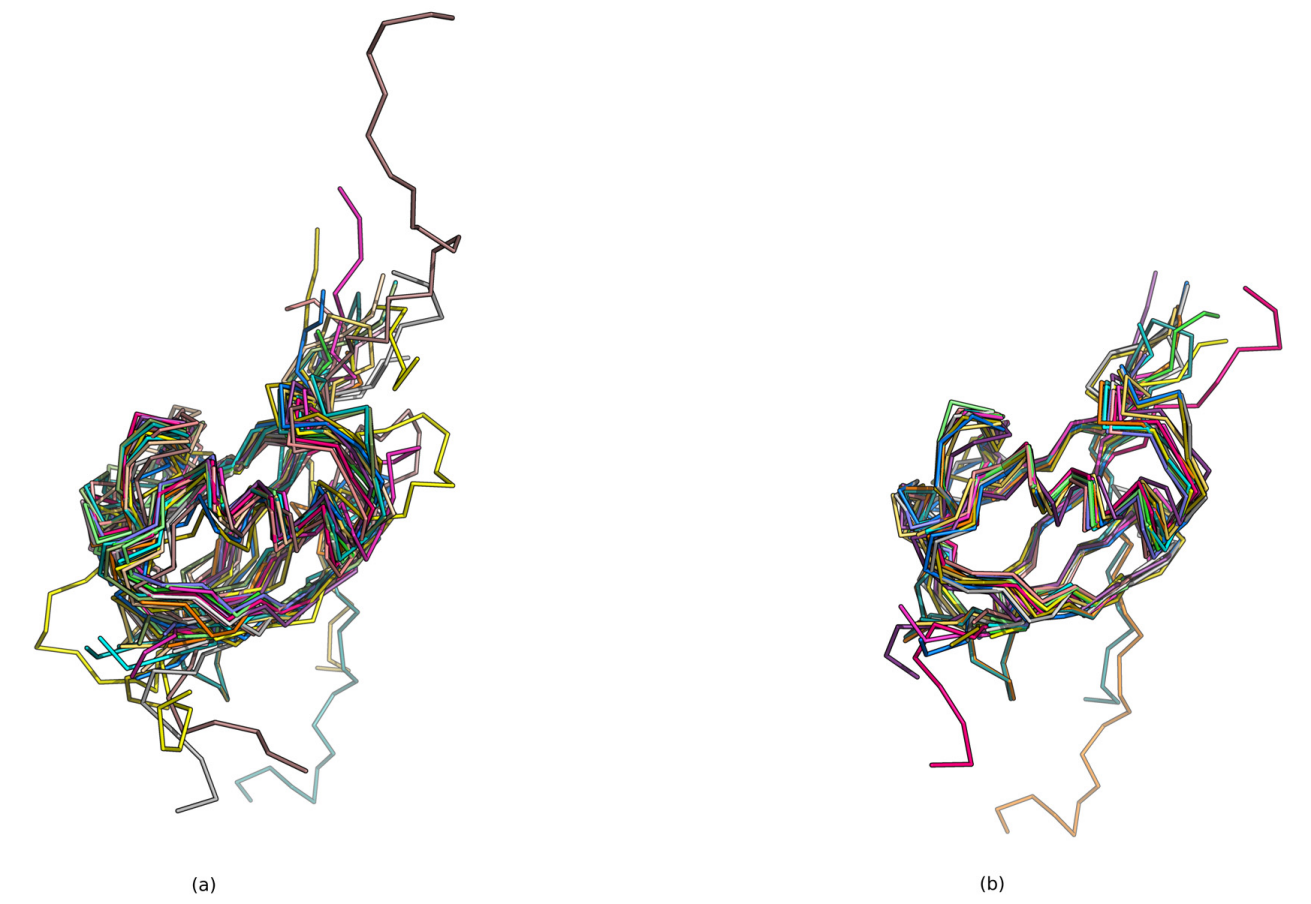
**Superposition of *β*-grasp query hits**. (a) Superposition of the top 20 hits to the *β*-grasp query structure d1ubia_. This superposition is generated by simply finding the orthogonal transformation that minimizes the RMSD between the C_*α *_atoms in the most central residues of the SSEs that are matched between the query and the hit structure. (b) Multiple alignment of the top 20 hits generated with MUSTANG [2]. Five structures are missing as they have multiple NMR models, which MUSTANG cannot currently handle. Figures were generated with PyMOL [63].

Figure 8(a) shows the superposition of the top 20 hits from the *β*-grasp query on the query structure (d1ubia_), showing that these high scoring matches are correctly matching corresponding SSEs in similar structures. This is not a multiple alignment, but simply a superposition of each structure onto the query structure according to the SSEs matched by our method. Figure 8(b) is a true multiple alignment of the top 20 hits from our method, generated with MUSTANG [2].

**Figure 9:**
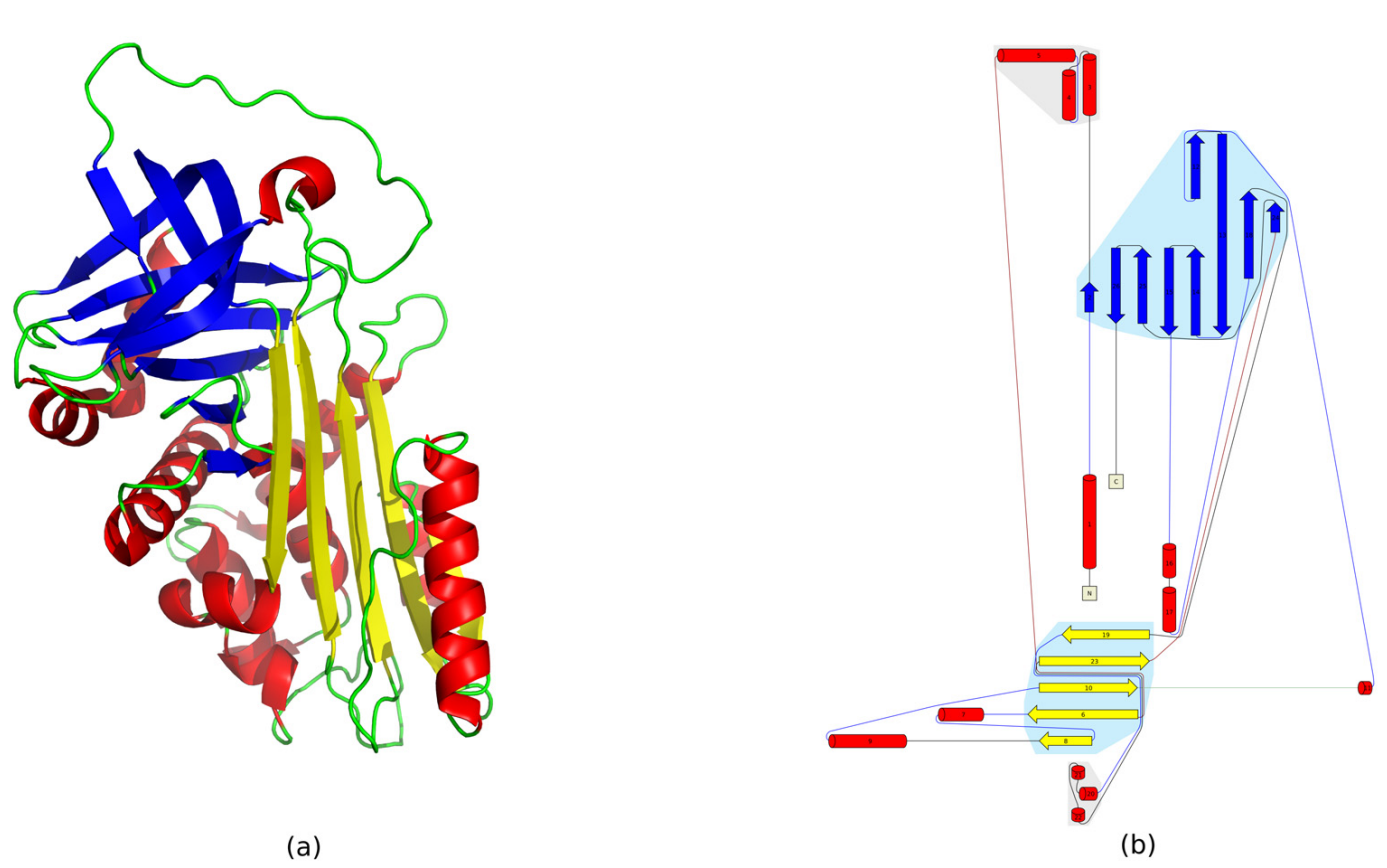
**Serpin B/C sheet query substructure**. (a) 3D structure of the canonical active serpin, *α*_1_-antitrypsin, PDB id 1QLP. Figure generated with PyMOL. (b) Topology cartoon of 1QLP. In both cartoons, the B/C sheet used as the substructure query is colored blue.

Substructure queries need not be entire structures themselves. In order to illustrate the ability of our method to find substructural matches, we chose the B/C sheet of the the canonical active serpin, *α*_1_-antitrypsin, PDB id 1QLP[42] as a query tableau. Figure 9 shows the substructure represented by this query. Of the total 18 structures in our database classified by SCOP as belonging to the serpin fold, 17 are matched as the top 17 hits (d1qlpa_ itself is the top hit). One, however, d1m93.1, is only at rank 1411 in the sorted hit list. This is the cleaved form of the serpin, but this does not account for the failure to detect its similarity, as, for example, d1jjo.1 is also the cleaved form and it is at rank 11 in the sorted hit list. Inspection of the tableaux for the B/C sheets of these two serpins shows that while that for d1jjo.1 is visibly similar to the query tableau, the relative angles of the strands in the B/C sheet of d1m93.1 are different enough from the query that the tableau is no longer sufficiently similar to find the correct matching of the two sheets. Using the entire serpin structure d1qlpa_ as the query, however, results in all 18 serpins as the top 18 hits.

**Figure 10:**
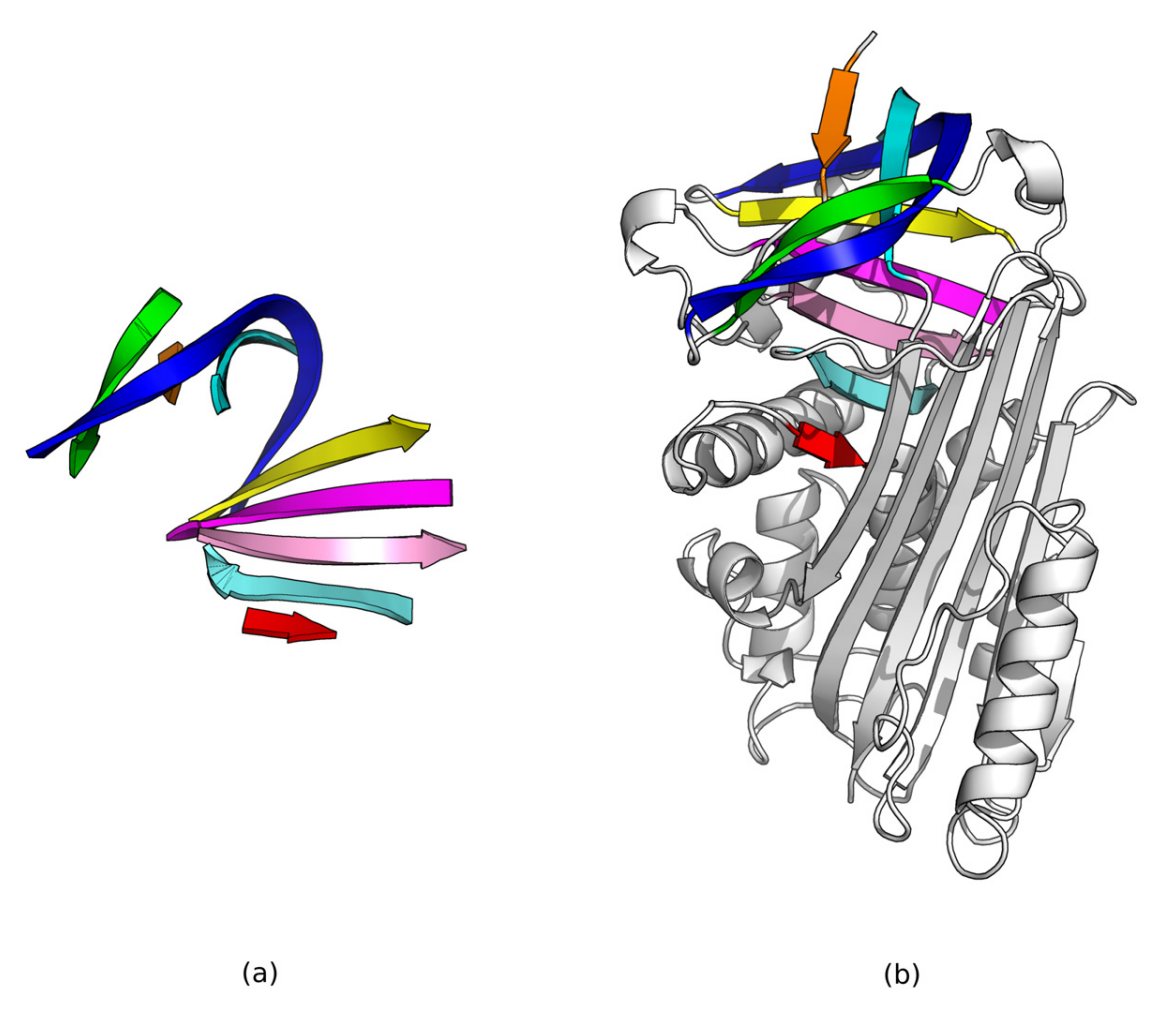
**The serpin B/C sheet query substructure found in a serpin from a thermophilic prokaryote**. (a) 3D cartoon of the strands in the serpin B/C sheet from PDB identifier 1QLP used as the query structure. (b) 3D cartoon of a serpin from a thermophilic prokaryote, SCOP identifier d1mtp.1, one of the top 10 hits to this query structure. SSEs from each structure that are found to be corresponding according to our method have the same color. Both cartoons were generated with PyMOL.

**Figure 11:**
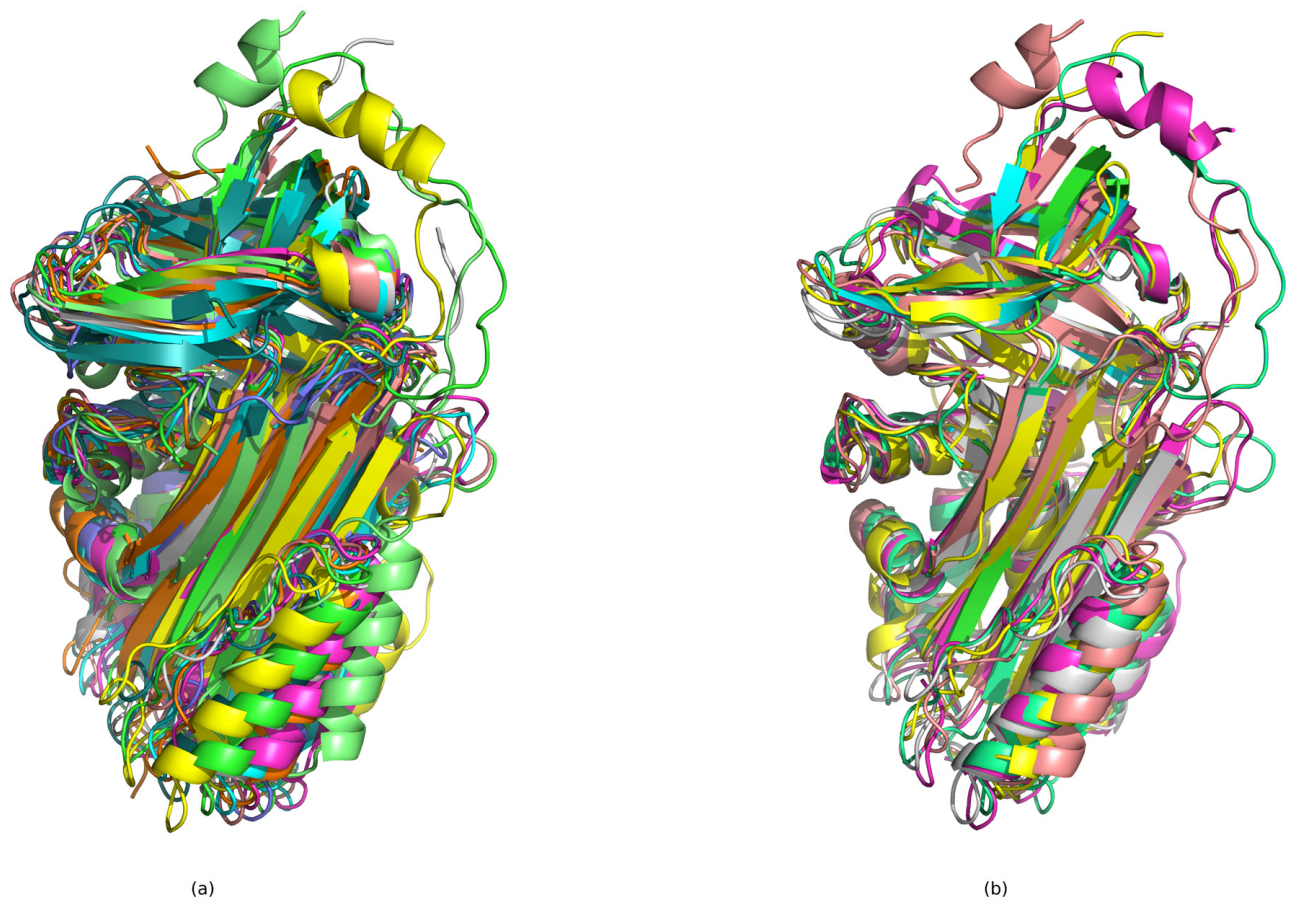
**Superposition of serpin B/C sheet query hits**. (a) Superposition of the top 10 hits to the serpin B/C sheet query substructure, generated by simple orthogonal transformation to minimize RMSD between C_*α *_atoms of the central residue in SSEs that are matched to each other between the query and the hit structure according to our method. (b) Multiple alignment of 5 of the top 10 hits generated by MUSTANG. Five are missing as they contain multiple chains which MUSTANG currently cannot process. Both images were generated with PyMOL.

Figure 10 shows the SSEs found as corresponding between the query substructure and a serpin from a thermophilic prokaryote, SCOP identifier d1mtp.1, at rank 7 in the sorted hit list. This demonstrates that the *β*-strands in the B/C sheet have been correctly identified by our method. Figure 11 shows superpositions and multiple alignments of the top 10 hits, showing that the B/C sheet has been correctly identified by our method in these serpins.

### Substructure search

Evaluated as a substructure (motif) query, the *β*-grasp query (d1ubia_) using the discrete tableau encoding has an AUC of 0.94. Since the data set used as the gold standard in this case is that defined by ProSMoS [16], that method by definition has an AUC of 1.00 on this query.

Table 7 shows the results of using the eight query structures to query the ASTRAL SCOP 1.73 95% sequence identity non-redundant database with ProSMoS, SSM [7], TOPS and our method. In all methods except ProSMoS, the exemplar SCOP structure is used directly as the query. ProSMoS, however, requires a manually edited query meta-matrix. We found that the query meta-matrices produced by the scripts included with ProSMoS applied to the query structures resulted in no hits, even when extensively edited to make them less specific, and so we used manually constructed meta-matrices instead (see Methods).

**Table 7:** Comparison of methods for substructure searching.

Fold	SCOP sid	P	S	T	Q	R	P/R	S/R	T/R	Q/R
*β*-grasp	d1ubia_	33	9	42	14	15	15/15	9/15	13/15	10/15
Key-barrel	d1tttb1	10	3	0	17	5	5/5	1/5	0/5	4/5
Immunoglobulin	d1ae6h1	27	1	4	1	11	9/11	1/11	4/11	1/11
Plait (ferredoxin)	d1bhne_	20	1	61	14	28	7/28	1/28	24/28	6/28
GFP-like	d1h6rb_	1	1	58	21	1	1/1	1/1	1/1	1/1
Jelly-roll	d2phlb1	1	1	19	15	12	1/12	1/12	10/12	5/12
TIM-barrel	d1tima_	16	16	40	33	32	16/32	16/32	28/32	32/32
NAD-binding fold	d1f6dc_	1	1	42	19	8	1/8	1/8	7/8	5/8

The number of superfamilies in the ASTRAL SCOP 95% sequence identity nonredundant database found for the eight query structures by ProSMoS (P), SSM (S), TOPS (T) and our method, QP tableau search (Q). ProSMoS and SSM return a list of significant hits, so the number of superfamilies found by these hits is shown. Other methods return scores for all database structures, and the number of superfamilies in the hits with a Z-score ≥ 3.0 are shown. The R column is the number of superfamilies, from the combined list of those found by all methods, in which the relevant query pattern or fold description is explicitly mentioned in the SCOP structure description. The ratio columns show the number of superfamilies in the R column found by the specified method.

Therefore the ProSMoS results reflect not only the performance of ProSMoS, but also our construction of the relevant query matrices.

We note that our results here differ significantly from those in Table 2 of [16]: our method of constructing this table is similar, but not identical, to that of [16], we have used slightly different queries (with the exception of the *β*-grasp query, where we used the meta-matrix described in [16]) and different versions of the software and a different database have been used. Consistently with [16], SSM finds the least number of matches. In our results, however, ProSMoS does not always return the greatest number of matches: sometimes TOPS does, since we are using a version of TOPS that computes scores for all matches, rather than the precomputed "classic" structure patterns.

TOPS also tends to have more false positives than ProSMoS or our method, that is, superfamilies found by TOPS that are not considered by the SCOP descriptions to contain the fold in question. This is consistent with TOPS being a purely topological method, which does not take account of other structural properties. Sometimes this also results in TOPS finding true positives which the other methods do not, for example when using the ferredoxin query, only TOPS finds the monooxygenase (hydroxylase) regulatory protein superfamily, d.137.1, which SCOP describes as having "some topological similarity to the ferredoxin-like fold". SCOP also notes in the family description for d.137.1.1 that "the solution structure determinations disagree in the relative orientations of two motifs", so topological similarity without taking into account more detailed structural similarity (specifically, SSE orientation, as used by our method and ProSMoS) is a more appropriate method to find matches to this structure, reflected in the relatively better performance of TOPS, and the previously discussed poor performance of our method on this query.

In order to better examine the unique properties of each method, Table 8 shows, for each method, the number of superfamilies found only by that method, and the number of these for which SCOP explicitly mentions the relevant query pattern. SSM finds no hits that the other methods do not, and TOPS usually finds the most. For the ferredoxin query, we can see that TOPS finds a large number of true positives (17) that the other methods do not; our method finds 3 and ProSMoS 1. However, on the TIM-barrel query, TOPS finds 11 unique hits, none of which are considered to contain the TIM-barrel motif according to SCOP, while our method finds 3 unique hits that all contain the TIM-barrel motif according to SCOP. With the exception of SSM, we can see that each method finds some unique true matches that the others do not. As an example of true positive hits that only our method finds, consider the jelly-roll query. Only QP tableau search finds the viral protein domain superfamily b.19.1, and the membrane penetration protein *μ*1 superfamily e.35.1, both of which are described by SCOP as containing a jelly-roll motif.

**Table 8:** Comparison of the unique hits from each method for substructure searching.

Fold	SCOP sid	Pu	Su	Tu	Qu	R	Pu/R	Su/R	Tu/R	Qu/R
*β*-grasp	d1ubia_	17	0	27	4	1	1/1	0/1	0/1	0/1
Key-barrel	d1tttb1	4	0	0	10	1	1/1	0/1	0/1	0/1
Immunoglobulin	d1ae6h1	25	0	2	0	9	7/9	0/9	2/9	0/9
Plait (ferredoxin)	d1bhne_	8	0	48	7	21	1/21	0/21	17/21	3/21
GFP-like	d1h6rb_	0	0	56	19	0	0/0	0/0	0/0	0/0
Jelly-roll	d2phlb1	0	0	15	11	9	0/9	0/9	7/9	2/9
TIM-barrel	d1tima_	0	0	11	3	3	0/3	0/3	0/3	3/3
NAD-binding fold	d1f6dc_	0	0	33	10	4	0/4	0/4	3/4	1/4

The number of superfamilies in the ASTRAL SCOP 95% sequence identity nonredundant database found by only that method for the eight query structures by ProSMoS (Pu), SSM (Su), TOPS (Tu) and our method, QP tableau search (Qu). The R column is the number of superfamilies, from the combined list of those found uniquely by each method, in which the relevant query pattern or fold description is explicitly mentioned in the SCOP structure description. The ratio columns show the number of superfamilies in the R column represented by the superfamilies found only by the specified method.

An interesting example is the ferredoxin fold, where the performance of our method as a structural search method is relatively poor. However, as a substructure search technique, some true positives are found only by our method. Only QP tableau search finds the peptide methionine sulfoxide reductase superfamily d.58.28, the CcmK-like superfamily d.58.56, and the release factor superfamily e.38.1. The first two are members of the ferredoxin-like fold but d.58.28 is described by SCOP as having the common fold "elaborated with additional secondary structures". The release factor superfamily (e.38.1) is described by SCOP as having 4 domains, one of which is a ferredoxin-like fold.

It is important to note several caveats in interpreting Table 7 and Table 8. First, as already discussed, ProSMoS queries were manually constructed, which is not the case for the other methods. Second, ProSMoS and SSM return a set of hits for a query, whereas the other methods return a matching score between the query and every database structure. Hence, in order to construct the tables, a cutoff score needs to be chosen (see Methods). The values in the tables are therefore very sensitive to the method used to choose the cutoff score: we could find arbitrarily many superfamilies simply by decreasing the value at which a score is considered a hit. Third, as discussed in [16], the lack of explicit mention of a structure in the SCOP description does not necessarily mean the structural motif is absent.

### Non-linear matchings

In order to verify the capability of our method to find non-linear matchings when the SSE ordering constraint is disabled, we first use an artificial test. Five different random permutations of the eight tableaux (that is, unique random re-orderings of the rows, and columns to preserve symmetry, of the tableaux) previously discussed and shown in Table 1 were generated. These were then used as queries, and the AUC for each calculated in the same way as for Table 1. The average AUC for each fold over its five permutations is shown in Table 9. From this table we can see that, despite the tableaux being permuted so that the SSEs in the query are no longer in the same sequence as in the database structures, the structures are still matched, albeit sometimes with a lower AUC than for the correctly ordered SSEs with the ordering constraint enabled.

**Table 9:** AUC for non-linear matchings averaged over five permutations of each of the fold query tableaux.

Fold	SCOP sid	Average AUC
*β*-grasp	d1ubia_	0.84
Key-barrel	d1tttb1	0.90
Immunoglobulin	d1ae6h1	0.92
Plait (ferredoxin)	d1bhne_	0.65
GFP-like	d1h6rb_	1.00
Jelly-roll	d2phlb1	0.90
TIM-barrel	d1tima_	1.00
NAD-binding fold	d1f6dc_	0.99

**Figure 12:**
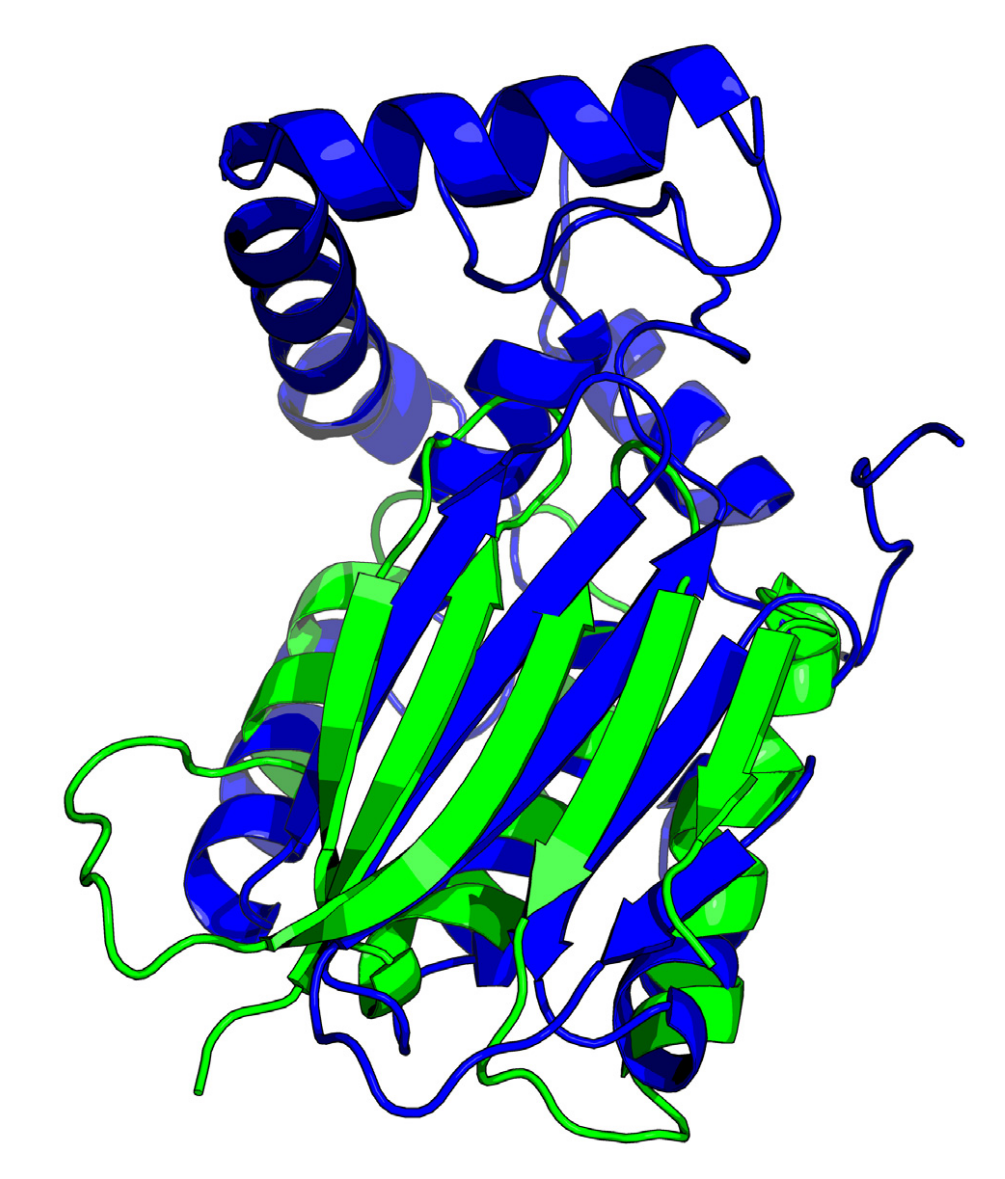
**Superposition of 2JMK and d1kb9b1_**. Superposition resulting from the non-linear matching of the query structure 2JMK (green) to the top hit d1kb9b1_ (blue). The figure was generated with PyMOL.

As a demonstration of a real case of a non-linear matching, we use as the query the hypothetical novel-fold protein TA0956 (PDB id 2JMK) [43] which was recently found to have several non-linear alignments [44]. When ordering constraints are enabled, our method finds no significant matches in the ASTRAL SCOP 1.73 95% sequence identity non-redundant database. However, when ordering constraints are disabled, some high-scoring hits are found, in particular the top-scoring hit is to d1kb9b1, cytochrome *bc*_1 _core subunit 2. The superposition of these two structures is shown in Figure 12. This is different from the result reported by Guerler and Knapp [44] using GANGSTA+ [44,45], who find that PDB identifier 1GO4 is the most similar structure (d1go4a_ is at rank 244 in the sorted hit list with our method). Therefore, we used the GANGSTA+ webserver [46] with 2JMK as the query, and found that after setting the selection criteria to require at least 10 matching SSEs, d1kb9b1 is the fourth most significant hit, demonstrating that GANGSTA+ is in agreement with our method that this non-linear matching is significant.

## Conclusion

We have introduced an improved method of searching for protein structures with similar folds using tableaux, incorporating constraints on the distances between SSEs to improve accuracy. This method is capable of finding either matches of an entire structure to the query, or matches where the query is a substructure of a larger structure. It is capable of finding non-linear matchings, where structurally equivalent parts do not have the same relative positions in the sequences of the two proteins. It also provides a set of corresponding SSEs, useful for manual validation of the result or as a seed for a more detailed structural alignment.

In assessing their VNS heuristic for MAX-CMO, Pelta *et al*. [34] ask whether it is necessary to solve MAX-CMO exactly in order to perform structure classification, and conclude that it is not: the heuristic solution is sufficient. We have shown that, consistent with previous work using the tableau representation of protein folds [18,20], the much more coarse-grained (and hence smaller and faster to solve) tableau representation is sufficient to accurately represent protein folds and perform structure classification. Specifically, we have shown that the efficient approximation of maximally-similar subtableaux extraction by relaxed quadratic programming is able to consistently classify folds at least as accurately as the VNS heuristic for MAX-CMO. In addition, our implementation is able to do so in less time than the MSVNS3 implementation described by [34].

We have demonstrated that the accuracy of our technique assessed as a protein fold recognition method compares favorably with some existing methods, and that it is fast enough to scan protein structure databases in a practical time, unlike the exact solution using CPLEX. It is, however, not as fast as some existing methods such as SHEBA and VAST, and the TableauSearch dynamic programming approximation introduced by [18] is faster still. These methods, however, cannot be used to find substructures or non-linear matchings.

We have also demonstrated the use of our technique as a method for searching for substructures in protein structures, and compared it with some existing techniques, including ProSMoS. Complications in objectively assessing the performance of these methods make definite conclusions in this area difficult: we can perhaps say at most that each method has different enough properties that they are all capable of finding unique hits that others miss. A structural biologist searching for matches to a motif or substructure, then, would do well to employ several of these methods rather than relying on just one. As noted by Li *et al*. [24], further theoretical work to find tight sufficient conditions for the QP to have an integer solution is required, although empirically an integer solution is almost always found.

## Methods

We built a database of tableaux, which is a file containing the tableau representation for each structure in the database. By pre-computing the tableaux in this way, only the query structure needs to have its tableau built when searching for occurrences of that structure. The search procedure is then to compute a matching score between the query tableau and each tableau in the database. Sorting the results by score allows the desired balance of sensitivity and specificity to be found by choosing a threshold score above which a match is considered a "hit" of the query to the database structure.

### Tableaux

An orientation matrix is a square symmetric matrix which describes the relative orientation of secondary structures in a protein; a tableau is a concise encoding of this matrix where the angles have been discretized using a double-quadrant encoding [19]. Tableaux have been found to accurately differentiate folds [20] and form the basis of the structural searching algorithm of [18].

The orientation matrix Ω, for a protein with *n *SSEs, is an *n *× *n *symmetric matrix. Each element *ω*_*ij *_of Ω, -*π *≤ *ω*_*ij *_≤ *π*, 1 ≤ *i*, *j *≤ *n *is the relative angle between the axes of SSEs *i *and *j*. Computing Ω therefore consists of three steps: defining the SSEs, fitting axes to the SSEs, and computing the interaxial angle between each pair of SSE axes.

The tableau is derived from the orientation matrix by a double-quadrant encoding scheme, in which the range of angles is divided into quadrants in two ways which differ in orientation by *π*/4, in order to prevent a small variation in angle resulting in two completely different encodings. The first quadrant encoding is labelled P, O, L, R for parallel, anti-parallel, crossing-left, and crossing-right, respectively, and the second arbitrarily E, D, S, T [19].

**Figure 13:**
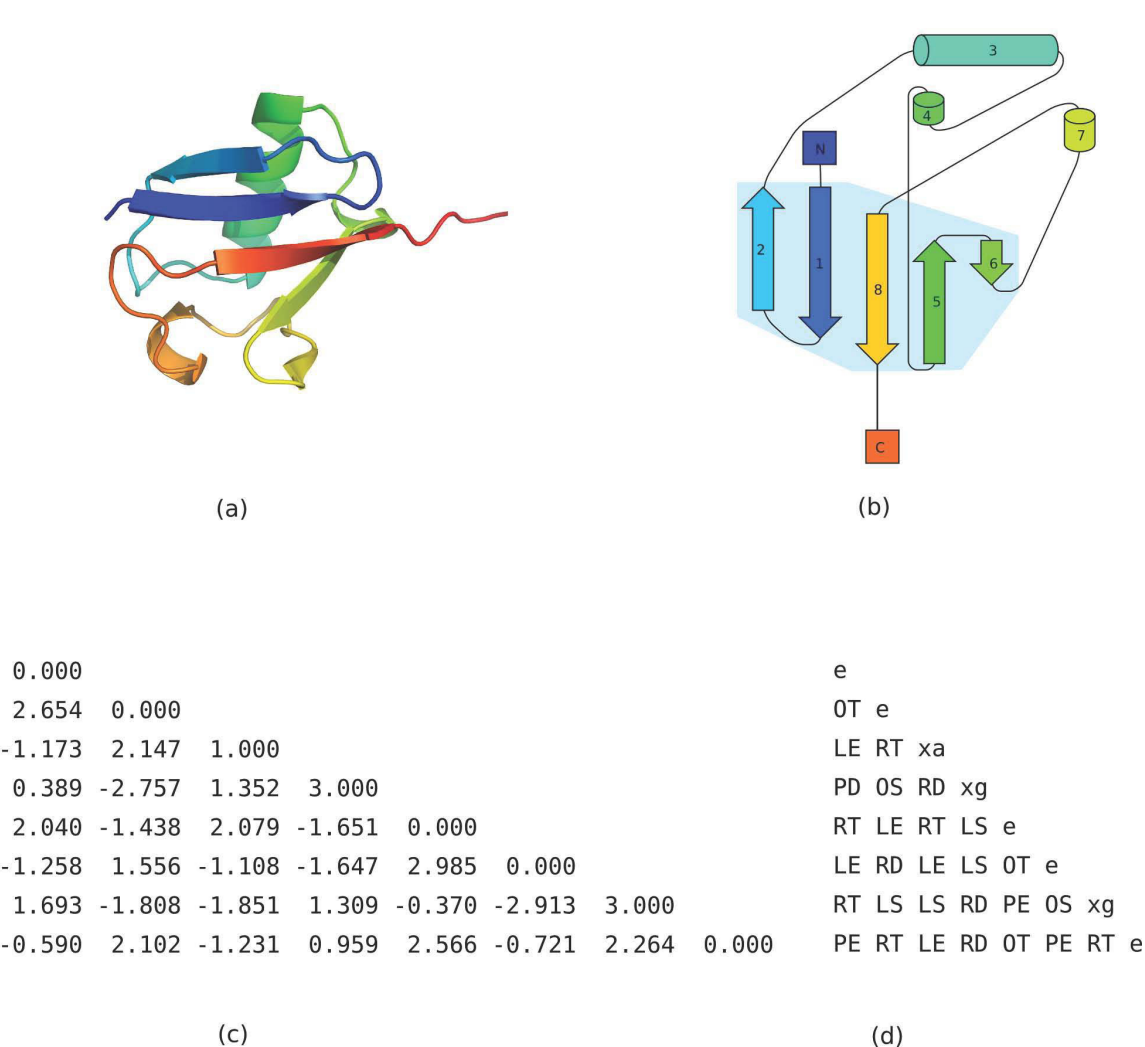
***β*-grasp query structure and tableau**. (a) 3D structure of ubiquitin, PDB identifier 1UBI. Image generated with PyMOL. (b) Topology cartoon of 1UBI. (c) Orientation matrix Ω for 1UBI. Each angle is in radians between -*π *and *π*. The main diagonal denotes the SSE type by 0.000, 1.000, 2.000, or 3.000 for *β*-strands, *α*-helices, *π*-helices, and 3_10_-helices, respectively. (d) Tableau for 1UBI. The main diagonal denotes the SSE type by e, xa, xi, or xg for *β*-strands, *α*-helices, *π*-helices, and 3_10_-helices, respectively.

Because the orientation matrix and tableau are symmetric, we need only store one triangle, and since the main diagonal is the meaningless self-angle, we use it to store the type of SSE represented by that row and column (see Figure 13).

### Quadratic integer programming formulation of extraction of maximally-similar subtableaux

The extraction of maximally-similar tableaux by quadratic integer programming (QIP) was described by Konagurthu *et al*. [18]. We use the same formulation:

Let Ω_*A *_= (), 1 ≤ *i*, *j *≤ *N*_*A *_be the orientation matrix for protein/structure A with *N*_*A *_SSEs and Ω_*B *_= (), 1 ≤ *i*, *j *≤ *N*_*B *_the orientation matrix for protein B with *N*_*B *_SSEs. Similarly let *T*_*A *_= () and *T*_*B *_= () be tableaux.

Define Boolean variables *x*_*ij*_, 1 ≤ *i *≤ *N*_*A*_, 1 ≤ *j *≤ *N*_*B *_where *x*_*ij *_= 1 indicates that the *i*th SSE in structure A is matched with the *j*th SSE in structure B.

Define a scoring function *ζ *which gives high scores to similar orientations, as follows:

(1)

where

(2)

When comparing (discrete) tableaux, the scoring function is defined as:

(3)

where  means the two tableau codes are identical, and  means they differ in only one quadrant, for example OS and OT, or OT and RT.

Then the QIP is:

maximize

(4)

subject to

(5)

(6)

Constraints (5) and (6) ensure each SSE in one tableau is matched with at most one SSE in the other. We introduce a further condition that two SSEs of different types (for example an *α*-helix and a *β*-strand) should not be matched, by assigning a low score to such a matching, for which we use the SSE type information encoded on the diagonal of the tableau or Ω matrix.

We may optionally avoid non-linear matchings by assigning a low score to matches between SSEs whose indices *i, k *in one structure and *j, l *in the other satisfy both of the following inequalities:

(7)

(8)

Without this condition, non-linear matchings are found.

In order to avoid false positives when SSEs in two structures have similar orientations relative to other SSEs in their respective structures, but are at very different distances from those other SSEs, we introduce a distance difference constraint, disallowing matches between SSEs where the difference in distances between the SSEs exceeds a threshold distance *τ*:

(9)

where *D*^*A *^= (), 1 ≤ *i*, *k *≤ *N*_*A *_and *D*^*B *^= (), 1 ≤ *j*, *l *≤ *N*_*B *_are SSE midpoint distance matrices. These are square symmetric matrices, of the same dimensions as the orientation matrices and tableaux, where each entry is the distance (in Ångströms) between the centroids of the C_*α *_atoms used in computing the respective SSEs' axes. We use the value *τ *= 4.0 Å for the distance difference threshold. This value was found empirically to give good results after testing various values between 2.0 Å and 8.0 Å on a subset of the queries in Table 1. As with tableaux, these distance matrices are precomputed and stored as a triangle with SSE information on the main diagonal. As before, we do not implement this constraint directly, but instead penalize the objective function when it is violated.

### Relaxed quadratic programming formulation and solution by interior point method

The QIP just described is NP-hard. Even though the instances are quite small, direct solving with CPLEX is too slow for practical use in searching a structure database [18]. A solution to this problem is provided by the work of Li *et al*. [24], whose formulation of biological network alignment is strikingly similar to the QIP for extracting maximally-similar tableaux. They show that the constraints (5)–(6) are totally unimodular, allowing the QIP to be relaxed to a quadratic program (QP) by removing the integrality constraints on the Boolean variables *x*_*ij*_, and that the QP will have an integer solution under certain conditions. This allows this (nonconvex) QP to be solved with an efficient interior ellipsoidal trust region method [47-49].

The standard form of a QP is

(10)

(11)

where *Q *is the symmetric *n *× *n *objective matrix, *A *is the constraint left-hand *m *× *n *matrix, *b *is the constraint right-hand *m *× 1 vector, *c *is the objective *n *× 1 vector, and *x *is the solution *n *× 1 vector.

In expressing the maximally-similar subtableaux QIP (4)–(6) in standard form, the vector *c *is zero as there is no linear term in the QIP objective function (4). The coefficient matrix *A *contains only 1s and 0s, since the constraints (5) and (6) are all of the form ; hence *A *is totally unimodular as shown by Li *et al*. [24]. The objective matrix *Q *contains the values of the scoring function *ζ*; these values are simply negated to transform the maximization problem (4) to the minimization problem (10).

Constraints (5) and (6), to ensure each SSE in one tableau is matched with at most one SSE in the other, are expressed in the standard form, that is, in the *A *matrix and *b *vector in equation (11). Constraints on SSE midpoint distance differences, mismatching SSE types and linearity of matchings, when desired, are not expressed directly, due to the infeasibly large matrices that would result from so doing. Rather, we relax them and penalize their violation by assigning a low score to such matches. This results in the following modified objective function for the discrete (tableau) version, where the negation to transform the problem to a minimization problem has also been shown:

(12)

The choice of 0 and 1 as the penalties in conjunction with the discrete tableau scoring function *ζ *(3) was found empirically to give good results.

We find in common with Li *et al *[24], that although the sufficient conditions described in the Supplementary Materials of [24], are not always met, that nevertheless an integer solution is almost always obtained.

### Evaluation

We computed tableaux for all 15273 domains in the 95% sequence identity non-redundant subset of the ASTRAL SCOP 1.73 database [14,29]. Unless otherwise stated, all queries, other than those for comparison with MAX-CMO using the Fischer or Nh3D data sets, discussed in the results were against this database of tableaux.

The larger scale query set is a set of 200 queries chosen from the ASTRAL SCOP 1.73 95% sequence identity non-redundant data set. The queries were chosen so that each class (*α*, *β*, *α*/*β*, *α *+ *β*) is represented in the query set in the same ratio as it is in the database. The list of queries is available with the source code and other data as described in the Availability section.

The Fischer data set, described in Table 2 of [35], consists of 68 proteins. Several PDB identifiers in this table have since been obsoleted, and we replaced these with their new versions according to the RCSB PDB website [50,15]. As was done by [34], we performed an all-against-all comparison in this data set, including redundant comparisons, resulting in 4624 comparisons.

The Nh3D v3.0 data set [36] consists of 806 structures, each representing a different CATH [37] topology. We performed the same 58838 comparisons as [34] by comparing each of the 73 structures listed in the Supplementary Material of [34] against every structure in the Nh3D v3.0 data set.

The MSVNS3 implementation and tableau search implementations produce unnormalized scores. MSVNS3 provides an overlap value, QP tableau search provides the maximum value of the tableau scoring function, and TableauSearch also provides an approximation of the maximum value of the tableau scoring function. For comparing sets of pairwise scores between proteins of different sizes, a normalization function is required. We use the same three normalization functions as [34], namely:

(13)

(14)

(15)

where score is the overlap value or tableau matching score for MSVNS3 or tableau search, respectively, and size is the number of contacts or number of SSEs for MSVNS3 or tableau search, respectively.

We evaluated the accuracy of structural search by counting a hit (a score above the threshold) as correct (a true positive) if the structure is in the same SCOP fold as the query structure, and incorrect (a false positive) otherwise. By using SCOP as the gold standard in this way, large scale automatic evaluation on a large number of different queries is possible.

For the Fischer data set, we evaluated at both the fold and class level. At the fold level, a true positive is counted when the score is above the current cutoff and the two structures are in the same fold according to Table 2 of [35]; similarly for the class level. For the Nh3D data set, we evaluated at both the architecture and class levels in CATH. At the architecture level, a true positive is counted when the score is above the current cutoff and the two structures have the same CATH architecture identifier and the same CATH class identifier. At the class level, they need only have the same class identifier.

Evaluation of the accuracy of substructure queries is more challenging, since we require as our gold standard a database of structures that contain a motif as a substructure. By using d1ubia_, an exemplar of the *β*-grasp fold, as the query, we used the data from Table 1 of [16] as the gold standard. A hit is considered a true positive if it is in the same SCOP superfamily as the exemplars listed in Table 1 of [16] for the *β*-grasp core and gregarious fold [51] categories, or if it is one of the structures considered by [16] to contain the *β*-grasp motif by structural drift [52].

We can then compute the true positive rate (*TPR*), or sensitivity, as



where *TP *is the number of true positives and *N *is the number of structures that match the query according to the gold standard (SCOP or the *β*-grasp data set). The false positive rate (*FPR*), which is equal to 1 – specificity, is



where *FP *is the number of false positives and *TN *is the number of true negatives. We then construct a ROC curve by plotting the *TPR *against the *FPR *for all values of the score threshold. The area under the ROC curve (*AUC*) is an overall measure of the quality of a classification method; a perfect classifier has *AUC *= 1.0, and a random classifier has *AUC *= 0.5. We approximate *AUC *by the trapezium integration rule.

When multiple queries are being assessed in one ROC curve, as in the Fischer and Nh3D data sets, and the 200 query set in the 95% sequence identity non-redundant subset of the ASTRAL SCOP 1.73 database, all the scores are combined together (after normalization), with each labelled as either a positive or a negative according to the appropriate gold standard. The ROC curves were then plotted with the ROCR package [53] in R [54] and the AUC and its standard error, when reported, are calculated by the Hanley-McNeil method [55].

For comparisons with other methods, SHEBA version 3.1.1, VAST downloaded from [56], ProSMoS downloaded from [57], and the TOPS matching software downloaded from [58] were used. TableauSearch was supplied by Dr Arun Konagurthu (personal communication). The authors' implementation of the VNS heuristic for MAX-CMO [34], MSVNS4MaxCMO, was downloaded from [59]. We used MSVNS3, the best performing version of the heuristic according to [34], for all tests.

For MAX-CMO, we generated contact maps for each structure with a threshold of 7.0 Å and sequence separation of 2 residues using a modified version of PConPy [60]. For QP tableau search, we generated tableaux and distance matrices for each structure with our own implementation of the tableau creation algorithm, including *π *and 3_10 _helices and using DSSP to define secondary structure elements. We built the TOPS database for the ASTRAL SCOP 1.73 95% sequence identity non-redundant subset using TOPS downloaded from [61] (July 2007) with default parameters (DSSP is used to define SSEs).

For the comparison with SSM, the SSM webserver [62] running SSM v2.36 and searching the SCOP 1.73 database was used, with default parameters. The search was restricted to the 95% sequence identity non-redundant subset by uploading the relevant ASTRAL SCOP identifier list as the list of SCOP 1.73 codes for the target.

For the comparison with ProSMoS, we found that the query meta-matrices produced by the scripts included with ProSMoS applied to the query structures resulted in no hits, even when extensively edited to make them less specific. Therefore, we manually constructed the query meta-matrices based on the following information:

• DSSP SSE assignments

• automatically generated topology cartoons

• 3D structure as shown by PyMOL [63]

• SCOP description of the fold

• the list file generated by the ProSMoS matrix generation scripts for the query structure.

When comparing methods for substructure search, apart from the detailed analysis of occurrences of the *β*-grasp motif available in Table 1 of [16], for constructing the comparisons detailed in Table 7 and Table 8 we follow a procedure similar to that described for Table 2 of [16], and count SCOP superfamily and fold descriptions that mention the query structure in question. ProSMoS and SSM give a list of hits to the query structure, and so we can simply count the number of superfamilies represented by these hits. Our method, however, and also the version of TOPS we are using, does not return such a list of hits but rather assigns a score to each database structure. This makes the computation of ROC curves, as previously described, a useful and appropriate method of assessment, but creates a difficulty for the superfamily counting method: we need a method to determine a score above which a hit is considered significant. In order to do this, we compute a Z-score for each matching of a query to a database structure as

(16)

where *s *is the score assigned to the matching, *μ *is the arithmetic mean of the scores for all database structures for that query, and *σ *is the standard deviation. For both our method and TOPS, we choose a significant hit to be a matching with *Z *≥ 3.0, a value which was found empirically to give a reasonable number of hits without excluding too many true positives amongst our set of example queries.

### Implementation

We implemented scripts for creating tableaux, building the tableaux database, evaluating results against SCOP and converting search output for visualization with PyMOL in Python. Our implementation of the tableaux creation algorithm optionally allows a list of SSEs in the structure to be represented in the tableau, rather than all SSEs in the structure, in order to generate tableaux for substructure queries. We used the BioPython library [64] and the Bio.PDB file parsing and structure class [65] to parse PDB files and the Bio.SCOP interface [66] to read SCOP and ASTRAL data. We re-implemented the QP solving algorithm [47,48], originally implemented in MATLAB [67], in Fortran 77 with the BLAS [68] and LAPACK [69] libraries for dense matrices, and the UMFPACK 5.2 [38-41] library for sparse matrices. The tableau searching program itself was written in Fortran 77.

## Availability

Source code, data sets, and executable binaries are available from .

## Authors' contributions

All authors contributed to the algorithm and evaluation design. AS implemented the algorithm and evaluation software, performed the tests, and prepared the manuscript and figures. All authors read and approved the final manuscript.
